# A novel role of ER stress signal transducer ATF6 in regulating enterovirus A71 viral protein stability

**DOI:** 10.1186/s12929-018-0412-x

**Published:** 2018-01-31

**Authors:** Jia-Rong Jheng, Kean-Seng Lau, Yueh-Wen Lan, Jim-Tong Horng

**Affiliations:** 1 Department of Biochemistry and Molecular Biology, College of Medicine, Chang Gung University, 259 Wen-Hwa First Road, Kweishan, 333 Taoyuan, Taiwan; 2 Research Center for Emerging Viral Infections, College of Medicine, Chang Gung University, Kweishan, 333 Taoyuan, Taiwan; 3 Molecular Infectious Disease Research Center, Chang Gung Memorial Hospital, 333 Taoyuan, Taiwan; 4 Research Center for Chinese Herbal Medicine and Research Center for Food and Cosmetic Safety, College of Human Ecology, Chang Gung University of Science and Technology, 333 Taoyuan, Taiwan

**Keywords:** ATF6, Enterovirus A71, Endoplasmic reticulum stress, Viral protein stability, Unfolded protein response, Viral replication

## Abstract

**Background:**

Due to limited coding capacity of viral genome, enterovirus A71 (EV-A71) co-opts host nuclear proteins for its replication. Upon ER stress, the ER-localized 90 kDa activating transcription factor 6 (p90ATF6) is proteolytically cleaved to produce the transcriptionally active amino-terminal 50 kDa (p50ATF6) product where it enters the nucleus to activate a subset of unfolded protein response and ER-associated degradation (also known as ERAD) genes. During EV-A71 infection, however, this p50ATF6 product was not detected in the nucleus, and its downstream target genes were not activated.

**Methods:**

We examined the role of ATF6 during EV-A71 infection, including its cleavage process and its role in viral life cycle by silencing or overexpressing ATF6.

**Results:**

We showed that a potential cleavage in the middle of p90ATF6 produced an amino-terminal ~ 45 kDa fragment in a viral protease-independent but EV-A71-dependent manner. The disappearance of ATF6 was not restricted to a specific strain of EV-A71 or cell type, and was not simply caused by picornavirus-mediated global translational shutoff. This cleavage of ATF6, which was most likely mediated by the host response, was nevertheless independent of both cellular caspases and XBP1-associated proteasomes. The silencing of ATF6 expression by small interfering RNA suppressed viral titers due to reduced viral protein stability. This effect was markedly restored by the ectopic expression of p90ATF6.

**Conclusion:**

Our findings indicate that ATF6 plays a distinct role in viral protein stability and that the host uses different cleavage strategies, rather than conventional cleavage by generating p50ATF6, to combat viral infection.

**Electronic supplementary material:**

The online version of this article (10.1186/s12929-018-0412-x) contains supplementary material, which is available to authorized users.

## Background

Disturbance of endoplasmic reticulum (ER) homeostasis can cause ER stress, which subsequently activates unfolded protein response (UPR) signaling pathways to alleviate the stress so that the cell either survives or undergoes apoptosis. Three transmembrane ER stress sensor proteins have been identified and comprehensively characterized: PKR-like ER kinase (PERK), activating transcription factor 6α (ATF6α), and inositol-requiring enzyme 1 (IRE1). These three proteins control three arms of the UPR signaling pathway and activate different subsets of genes to maintain ER homeostasis. The activation of PERK initiates the phosphorylation of eukaryotic initiation factor 2α (eIF2α), resulting in the inhibition of global translation [3]. IRE1 splices transcription factor X-box-binding protein 1 (XBP1) mRNA, leading to a frameshift and the subsequent translation of functional XBP1 protein [28]. By binding the ER stress response element (ERSE) and UPR element promoter regions, the spliced transcription factor XBP1 can sequentially stimulate different subsets of genes encoding proteins that promote the folding and degradation of misfolded/unfolded proteins in a process termed ER-associated degradation (also known as ERAD) [17].

ATF6α is a type II ER membrane glycoprotein whose N-terminus is exposed in the cytoplasm. ATF6α is a 90 kDa protein of 670 amino acids and thus frequently designated as p90ATF6. ATF6α comprises several functional domains: a basic leucine zipper, a transcriptional activation domain (TAD), Golgi localization sequences (GLS), and cleavage sites for site-1 and site-2 proteases (S1P and S2P, respectively). ATF6 is retained in the ER through interactions between its luminal tail and the ER chaperone BiP/GRP78 [2, 18, 19]. Upon activation, ATF6 dissociates from BiP to expose its GLS, and it is translocated to the Golgi apparatus, where it is cleaved by S1P and S2P to release its N-terminal transcriptionally active 50 kDa fragment (p50ATF6) [2, 27]. The cytosolic p50ATF6 is then transported to the nucleus, where it activates the transcription of a collection of ER stress target genes, including BiP, by binding to a consensus ERSE sequence in the promoter regions of these proteins [4, 14].

Enterovirus A71 (EV-A71) is a member of the family Picornaviridae. The 7.5 kb (+)RNA genome of EV-A71 encodes a polyprotein that is proteolytically cleaved into distinct proteins by the virus-encoded proteases 2A^pro^ and 3C^pro^ [12, 16]. The structural proteins VP1 to VP4 and the nonstructural proteins 2A to 2C and 3A to 3D are required for viral replication [16]. Picornaviral proteases perform self-cleavage, which is necessary for the production of functional viral proteins, and they cause a global shutoff of host protein synthesis at the transcriptional and translational levels. Inhibition at the transcriptional level involves several transcription factors, such as TATA-binding protein and cyclic AMP-responsive element-binding protein (as known as CREB), which are cleaved by 3C^pro^ to regulate cellular RNA synthesis [23]. Host eIF4G, which is involved in cap-dependent translation, is mainly cleaved by 2A^pro^ to downregulate host translation [10].

Various studies have suggested that there is an association between the ER stress response and viral infection, and many viruses have been shown to regulate the UPR to benefit their replication or to trigger the immune response [6]. These viruses include some flaviviruses and enteroviruses [1, 8, 9, 20–22, 30]. We previously demonstrated that EV-A71 infection activates and modifies the UPR signaling pathways to benefit viral replication [7]. The ATF6 pathway is presumably activated during viral infection, as suggested by the proteolytic cleavage of p90ATF6, whereas the production of the active p50ATF6 fragment and the activation of its downstream target gene BiP in an ATF6- and ERSE-dependent manner do not occur [7]. In the present study, we further examined the role of ATF6 during EV-A71 infection, including its cleavage process and its role in viral life cycle.

## Methods

### Cell culture and viral infection

RD, MCF7, and HEK293T cells were obtained from ATCC and cultured in Dulbecco’s modified Eagle’s medium (DMEM) supplemented with 10% fetal bovine serum (FBS) under humidified 5% CO_2_. EV-A71 BrCr was obtained from the ATCC (accession no. VR 784), and EV-A71 strain 1743 and echovirus 9 were obtained from Chang Gung Memorial Hospital, Linkou, Taiwan. EV-A71 strain 4643 was a gift from Dr. Jen-Ren Wang, National Cheng Kung University, Tainan, Taiwan [26]. EV-A71 strain 2231 was amplified from a full-length infectious clone of EV-A71 (TW/2231/98) in pCR-XL-TOPO (Invitrogen, Carlsbad, CA), which was obtained from Dr. Meishan Ho of Sinica Academia, Taipei. Viral propagation was performed in RD cells [25]. For the plaque assay, RD cells were adsorbed with EV-A71 for 1 h (from - 1 to 0 h postinfection (p.i.)), and the unbound virus was removed after adsorption (0 h p.i.) by two washes with phosphate-buffered saline, after which the cells were overlaid with DMEM containing 2% FBS and 0.3% agarose. The cells were incubated at 37 °C for 4 days in an atmosphere of 5% CO_2_ and were then fixed with 4% formaldehyde for at least 2 h prior to crystal violet staining. The titer was recorded as the number of plaque-forming units per milliliter.

### Antibodies and reagents

Rabbit polyclonal anti-ATF6 antibodies raised against amino acids 6–307 of ATF6 were described previously [7]. Rabbit polyclonal antibodies against FLAG, CstF-64, and GFP were from Santa Cruz Biotechnology (Santa Cruz, CA, USA). Rabbit polyclonal antibodies against PARP (#9542) and eIF4G were from Cell Signaling Technologies (Beverly, MA, USA). Monoclonal antibodies against EV-A71 3C^pro^ and 3D were generously provided by Dr. Shin-Ru Shih of Chang Gung University, Taiwan [5, 24]. Monoclonal antibodies against HA (clone 3F10) and His tag were from Roche (Indianapolis, IN, USA) and Serotec (Oxford, UK), respectively. Antibody against GAPDH was from Abnova (Taoyuan, Taiwan). The sequence of small interfering RNA for ATF6 (siATF6) was 5′-UAC ACU UGU AGC UCA CUC CCU GAG U-3′, and that of siXBP1 was 5′-CUC AGA CUA CGU GCA CCU CUG CAG CA-3′. The caspase inhibitors Z-VAD-FMK (Cat. # 1140-1) and Q-VD-OPH (Cat. # 1170-1) were from BioVision.

### Plasmid construction

Full-length p90ATF6 was cloned into the yT&A cloning vector (Yeastern Biotech, Taipei, Taiwan) via reverse transcription from RD cells. The primers used in this study are listed in Additional file 1: Table S1. For HA-p90ATF6-mycHis construction, this ATF6 was reamplified using primers with a built-in HA sequence. This HA-ATF6 fragment was cloned into yT&A and was released with the restriction enzymes HindIII and NotI prior to religation into pcDNA3.1 B+ Myc/His to create the HA-p90ATF6-mycHis construct. Viral 2A, wild-type (Wt) 3C, and 3C containing point mutations were cloned in pLKO-AS3w-puro using primers listed in Additional file 1: Table S1. For the Wt ATF6 and its variants fused to HA and FLAG tags, the vector pLKO-AS3w-puro was first modified by adding a FLAG tag that was PCR amplified from pFLAG-CMV-5.1 (Sigma-Aldrich) using the primers 5′-CgC AAA Tgg gCg gTA ggC gTg-3′ and 5′-CCA CAg TTT AAA CCT ACT TgT CAT CgT CgT CC-3′. The PCR product was restriction digested with EcoRI/PmeI and was inserted into pLKO-AS3w-puro to produce pLKO-AS3w-puro-cFLAG. p90ATF6, ATF6 (1–516), p90ATF6 (G512A), p90ATF6 (G517A), and p90ATF6 (G512A/G517A) were cloned into pLKO-AS3w-puro-cFLAG; the primers are listed in Additional file 1: Table S1. The EV-A71 2231 IRES reporter (pcDNA-RHF 2231IRES) was constructed as a reporter for studying IRES-dependent translation by inserting the EV-A71 5′UTR upstream of the open reading frame of the firefly luciferase (FLuc) vector. The EV-A71 2231 strain 5′UTR cDNA fragments were produced from an EV-A71 infectious clone by PCR amplification using primers with BamHI and XhoI restriction sites (listed in Additional file 1: Table S1). The amplified DNA fragments were digested and then ligated to pcDNA-RHF, which comprised pcDNA3.1(+)/myc-His B containing Renilla luciferase (Rluc), a hairpin sequence, and FLuc.

### EV-A71 IRES activity assay

RD cells were transfected with scramble control ATF6 (scATF6) or siATF6 for 2 days, followed by the transfection of pcDNA-RHF 2231IRES or its vector control pcDNA-RHF for 24 h. Cell lysates were collected for the luciferase assay.

### Data analysis

The data were analyzed using two-tailed Student’s t-tests and are expressed as the means ± SD. *P* < 0.05 was considered statistically significant.

## Results

### Reduction in p90ATF6 was detected among different EV-A71 strains and enteroviruses and was not solely caused by picornavirus-mediated translational shutoff

**Fig. 1:**
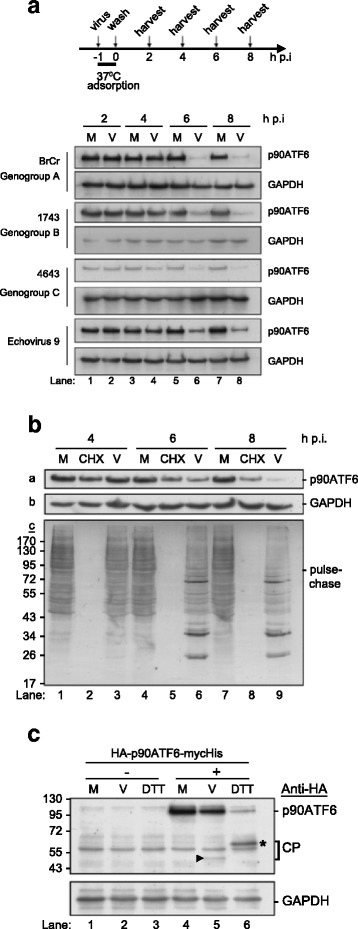
ATF6 cleavage was common among different picornaviruses. **a** RD cells were mock infected (M) or infected (V) with different strains of EV-A71 and echovirus 9 at an MOI of 10. As outlined in the upper panel, cells were infected with virus for 1 h at - 1 h p.i., after which the unbound virus was removed and replaced with DMEM containing 2% FBS at 0 h p.i. Cell lysates were harvested at 2, 4, 6, and 8 h p.i. and were evaluated by western blotting using an antibody specific for endogenous ATF6. Anti-GAPDH was used as an internal loading control. **b** Assessment of p90ATF6 level in EV71-infected cells by pulse–chase and immunoblotting assays. RD cells were infected with EV71 (strain 2231) or treated with (10 μg/ml) cycloheximide (CHX), and bulk proteins were metabolically labeled with [^35^S]methionine/cysteine for 1 h before harvest at the time points indicated. Cell lysates were collected and analyzed by SDS-10%PAGE and autoradiography (panel c). The basal level of p90ATF6 was detected by western blotting using antibodies against ATF6 (panel a). GAPDH was used to monitor equal loading (panel b). **c** EV71-infected p90ATF6-overexpressing RD cells were harvested at 7 h or were treated with 2.5 mM DTT for 7 h and evaluated by western blotting using an antibody specific for the HA tag. DTT-treated cells were used as a positive control for ATF6 cleavage under conditions of definitive UPR induction. The products of ATF6 cleavage induced by viral infection and DTT are indicated by an arrowhead and an asterisk, respectively

We asked whether the EV-A71-induced reduction in p90ATF6 was restricted to a specific strain of EV-A71 (Fig. 1a). RD cells were infected with different strains of EV-A71 corresponding to genogroups A to C. Cell lysates were harvested at the times indicated in Fig. 1a, and they were evaluated by western blot analysis using an antibody specific for ATF6. Infection with the prototype BrCr (genogroup A) reduced p90ATF6 beginning at 6 h p.i. compared with mock-infected cells (lanes 6 and 8 versus lanes 5 and 7). Interestingly, genogroups B and C, represented by strains 1743 and 4643, respectively, also reduced the expression of p90ATF6 with similar inhibition kinetics (Fig. 1a). This inhibitory effect was also observed with infection by another enterovirus, echovirus 9. This result suggests functional conservation among different EV-A71 strains and enteroviruses. However, we cannot exclude the possibility that enterovirus-activated cellular proteins play a role in p90ATF6 decrease. To determine whether the reduction in ATF6 by EV-A71 was cell type dependent, we infected the glioblastoma cell line SF268 with EV-A71 BrCr and strain 4643. The virus-induced p90ATF6 cleavage in SF268 cells displayed a pattern similar to that in RD cells (data not shown). Loss of the p90ATF6 signal was also observed in HEK 293 T (see below), SF268 (glioblastoma) and MCF7 (breast adenocarcinoma) cells (data not shown), implying that the disappearance of ATF6 was not cell type dependent.

Enterovirus infection attenuates cellular protein synthesis [16]. We performed pulse–chase experiments and western blotting to monitor the expression level of endogenous p90ATF6 (Fig. 1b). RD cells were infected with EV-A71 or treated with cycloheximide, and proteins were metabolically labeled with [^35^S]methionine/cysteine before being harvested at the times indicated in Fig. 1b (panel c). The respective basal level of ATF6 was detected by western blotting (panel a). Cycloheximide treatment exerted more effective translational shutoff than did virus infection at 4 and 6 h p.i. (lanes 2 and 5 versus 3 and 6, panel c), but the endogenous level of ATF6 was similar to that of the mock controls (panel a). Cycloheximide-treated cells had visible ATF6 at 8 h p.i. (lane 8, panel a), although newly synthesized proteins were not visible (lane 8, panel c). Virus infection caused a greater reduction in ATF6 level at this time. These results indicate that the disappearance of intact ATF6 during infection of cells with EV-A71 was not only caused by the EV-A71-induced translational attenuation; but also likely caused by EV-A71-induced cleavage (or degradation) of ATF6.

To determine whether the loss of p90ATF6 signal was the result of degradation or cleavage, we constructed a chimeric protein in which ATF6 was flanked by epitope tags to produce HA-p90ATF6-mycHis. This chimeric protein was similar to the wild-type protein in that it translocated into the nucleus through the cytoplasm when DTT-treated cells were examined by immunofluorescence microscopy using anti-HA antibodies (data not shown). In addition, the reduction in the level of p90ATF6 by DTT treatment correlated with the appearance of the p50ATF6 band, suggesting that the epitope-tagged ATF6 was functional (indicated by an asterisk in lane 6, Fig. 1c). The expression level of epitope-tagged p90ATF6 was reduced by viral infection, and a virus-specific fragment of ~ 45 kDa was detected by western blotting using an antibody specific for HA (indicated by the arrowhead in lane 5, Fig. 1c). This fragment, which was also detected with ATF6 antibodies (data not shown), was named p45ATF6. These findings suggested that ATF6 was cleaved rather than degraded by EV-A71 infection and the aberrant cleavage of ATF6 underlying this finding had not yet been explored.

### Viral titers were reduced in ATF6-silenced RD cells

**Fig. 2:**
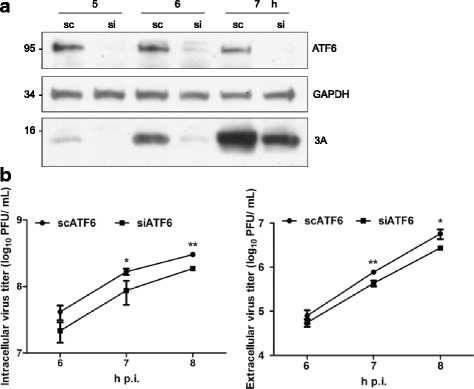
Viral titers were inhibited in ATF6-silenced RD cells. RD cells were transfected with 20 nM scATF6 and siATF6 for 3 days, followed by infection with EV-A71 (MOI of 10). Cells or culture supernatants were harvested at the indicated time points for viral protein quantitation (**a**) or viral titer determination (**b**). The data are representative of three independent experiments (**a**). The data are presented as the means ± SD. *n* = 3, *P < 0.05, and **P < 0.01

**Fig. 3:**
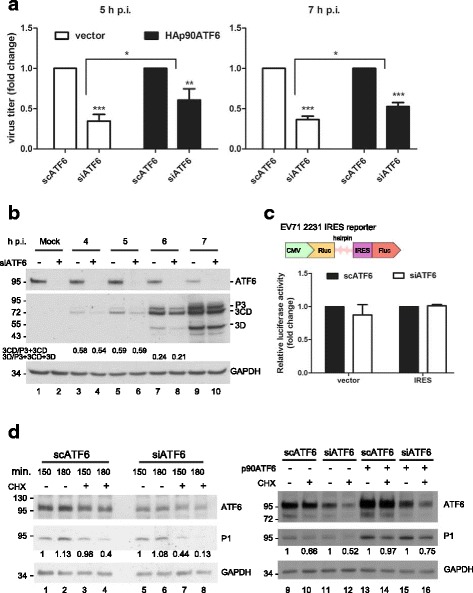
ATF6 is necessary and sufficient for viral replication by stabilizing P1 precursor viral protein. After transfection with scATF6 or siATF6 for 48 h, RD cells were transfected with HA-ATF6-FLAG (**a**) or HA-ATF6-FLAG and AS3w-P1-cFLAG (right panel, **d**). At 72 h after siRNA transfection, the cells were infected with EV-A71 at an MOI of 10. Cells were collected at the indicated time points for titer determination (**a**) and western blotting (**d**). **a** HA-ATF6-FLAG alleviated the siATF6-mediated reduction in viral production. The cells were collected and subjected to three freeze-thaw cycles for viral progeny collection. The ratio of viral titers in siATF6-transfected cells in the presence of HA-ATF6-FLAG or vector control was normalized to that of the scATF6-transfected cells, which was set arbitrarily at 1. The data are presented as the means ± SD. n = 3, *P < 0.05, **P < 0.01, and ***P < 0.001. **b** ATF6 was not associated with viral protein processing. Anti-ATF6 and anti-GAPDH antibodies were used to detect ATF6 and GAPDH. Anti-3D, which recognized both precursors (P3 and 3CD) and the mature viral protein 3D, was used to monitor the processing of the viral P3 region. The processing efficiency was expressed as a ratio of the band intensities of 3CD and (P3 + 3CD) (lanes 3-6) or of 3D and (P3 + 3CD + 3D) (lanes 7-8). The band intensity of 3D at 4 and 5 h p.i. was below the detection limit. The bands at 7 h p.i. were saturated and were not included in the analysis. Representative data from three independent experiments are presented. **c** ATF6 did not affect IRES activity. A structural diagram of the pcDNA-RHF EV-A71 IRES reporter is shown at the top. siRNA transfection was performed, and the 2231 IRES reporter plasmid and its corresponding control vector (pcDNA-RHF) were transfected 48 h later, followed by lysate preparation for a reporter assay. The relative luciferase activity of siATF6-transfected cells was normalized to that of scATF6 control-treated cells (arbitrarily set at 1). The data are presented as the means ± SD from three independent experiments. **d** ATF6 was associated with viral protein stability. CHX (10 μg/mL) was added 72 h after siATF6 transfection, and the cells were harvested for western analysis at 150 and 180 min (lanes 1-8) or at 150 min (lanes 9-16). Anti-VP1 and anti-ATF6 antibodies were used to detect P1 and endogenous and exogenous ATF6. The ratio of P1 to GAPDH was normalized to that at the 150 min time point in the absence of CHX in scATF6- or siATF6-transfected cells, which was set arbitrarily at 1. The data are representative of three independent experiments

To assess the role of ATF6 in viral replication, we silenced ATF6 by small interfering RNA to mimic the host’s effect of reducing the expression of ATF6 in the very beginning of the infectious cycle. The knockdown of ATF6 neither reduced cell viability (according to an MTT assay) nor affected the integrity of the ER, as demonstrated by the observation that the distribution of the ER protein calnexin was not altered by a cytoplasmic/microsome fractionation assay in ATF6 knockdown cells (data not shown). The viral protein synthesis was lower in the cells transfected with siATF6 than in the cells transfected with scATF6 (Fig. 2a). This inhibitory effect was reflected by a reduction in the viral titer (Figs. 2b and 3a). The extracellular viral titer was inhibited to a similar degree in the siATF6-transfected cells, indicating that ATF6 did not inhibit the release of progeny virus (right panel, Fig. 2b). The reduction in viral titer in the siATF6 cells was significantly ameliorated by the transfection of pLKO-AS3w vector containing HA-tagged ATF6 (HA-ATF6-FLAG), but not by the vector control (Fig. 3a). To further investigate the mode-of-action of ATF6 in viral protein expression, we examined viral protein processing (maturation), IRES-dependent translation, and viral protein stability in siATF6 cells (Fig. 3b-d). Figure 3b illustrates that a monoclonal antibody against 3D recognized not only the mature 3D but also the precursors P3 and 3CD in EV-A71-infected cells. Although viral protein expression was inhibited by silencing ATF6, the amounts of processed proteins relative to precursor proteins were barely altered in the siATF6 cells, indicating that the inhibition of viral protein shown in Fig. 2a was not a result of reduced processing (Fig. 3b). We next examined IRES activity using a bicistronic reporter in which the translation of the first cistronic RLuc was cap dependent and that of the second cistronic FLuc was cap independent (upper panel, Fig. 3c). The IRES activity, measured as the ratio of FLuc expression to RLuc expression, was not altered in the ATF6-silenced RD cells, suggesting that the reduced viral protein expression may not have resulted from defective IRES activity when ATF6 was silenced (right, Fig. 3c). ATF6 also did not affect cap-dependent translation in cells that were transfected with the reporter control (pcDNA-RHF), which translated RLuc cap-dependently (left, Fig. 3c). However, in the presence of cycloheximide (CHX), the stability of ectopic P1 precursor viral protein was markedly reduced in siATF6-transfected cells (0.98 in lane 3 versus 0.44 in lane 7 at 150 min and 0.4 in lane 4 versus 0.13 in lane 8 at 180 min, Fig. 3d). As expected, the parallel overexpression of HA-ATF6-FLAG apparently rescued the stability of P1 in scATF6- and siATF6-silenced cells (0.66 in lane 10 versus 0.97 in lane 14 in scATF6-transfected cells and 0.52 in lane 12 versus 0.75 in lane 16 in siATF6-transfected cells, Fig. 3d). The expression of virally encoded 3D was also elevated by HA-ATF6-FLAG in RD cells infected with EV-A71 (data not shown). Taken together, our data suggest that ATF6 plays an essential role in viral protein stability.

### Viral 3C^pro^ but not 2A^pro^ cleaved ATF6 in cultured cells

**Fig. 4:**
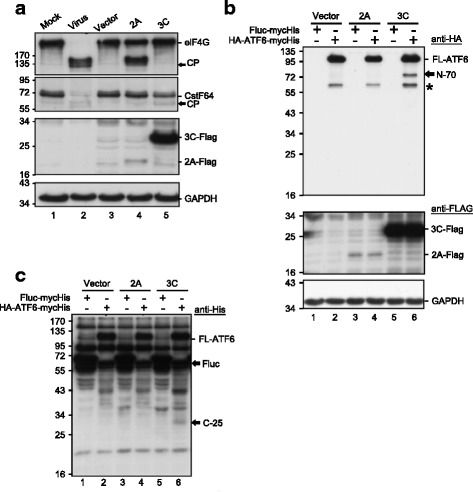
ATF6 was cleaved by viral-encoded proteases in HEK293T cells. The expression of recombinant 2A^pro^-FLAG and 3C^pro^-FLAG in the AS3w vector was detected with anti-FLAG antibodies, and GAPDH was used as a loading control. **a** Validation of the protease catalytic activity of recombinant viral 2A^pro^ and 3C^pro^ proteins. HEK293T cells were either infected with EV-A71 at an MOI of 200 as a positive control or were transfected with plasmids encoding 2A^pro^-FLAG or 3C^pro^-FLAG for 24 h. The cleavage of endogenous eIF4G and CstF-64, substrates of viral 2A^pro^ and 3C^pro^, respectively, was assessed by immunoblotting using antibodies specific for eIF4G and CstF-64. The cleavage products (CP) of eIF4G and CstF-64 are indicated by arrows. **b**, **c** ATF6 was cleaved by viral 3C^pro^ but not by 2A^pro^. HEK293T cells were first transfected with a plasmid encoding HA-ATF6-mycHis or a control plasmid encoding FLuc-mycHis (pcDNA3.1/mycHis B(+)::FLuc). After 24 h, the cells were transfected with a vector control or plasmid encoding 2A-FLAG or 3C-FLAG. The cells were harvested and lysates were prepared 24 h post transfection. Equal amounts of cell lysates (30 μg/lane) were analyzed with 10% SDS-PAGE, followed by immunoblotting using antibodies against HA (**b**) or His tag (**c**) to detect the N- or C-terminal region of recombinant ATF6, respectively. The asterisk denotes a nonspecific autoproteolytic product. The data shown here are representative of at least three independent experiments

**Fig. 5:**
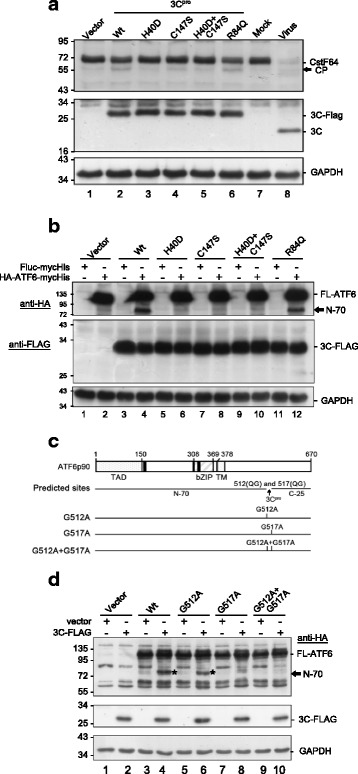
Cleavage of ATF6 by the proteolytic activity of 3C^pro^. **a** Examination of the protease catalytic activity of Wt and individual mutants of 3C^pro^. HEK293T cells (1 × 10^6^ cells per well in six-well plates) were transfected with the empty vector AS3w or with constructs carrying Wt 3C, the protease-deficient H40D or C147S single mutant, the H40D/C147S double mutant, or the R84Q RNA-binding mutant of 3C. Cell lysates were prepared 24 h post transfection and were subjected to SDS-PAGE and immunoblotting using antibodies against CstF-64, 3C, and GAPDH. A control viral infection was performed at an MOI of 200, and the cells were harvested at 16 h p.i. The cleavage products (CP) of CstF-64 are indicated by an arrow. **b** Cleavage of ATF6 by Wt or mutant 3C^pro^. HEK293T cells were first transfected with a plasmid encoding HA-ATF6-mycHis or the control vector pcDNA3.1/mycHis B(+)::FLuc. After 24 h, the cells were transfected with the control vector or plasmids encoding Wt or mutant 3C^pro^. Cells were harvested and lysates were prepared 24 h post transfection. Equal amounts of cell lysates (30 μg/lane) were analyzed with 10% SDS-PAGE, followed by immunoblotting using antibodies against HA, FLAG, or GAPDH. The data shown here are representative of three independent experiments. **c** Predicted cleavage sites of 3C^pro^ at the dipeptide glutamine–glycine (QG) near amino acids 512 and 517. Plasmids encoding point mutations (QG→QA) were constructed to generate the G512A, G517A, and G512A/G517A mutations of HA-ATF6-mycHis. **d** Mapping of the cleavage sites. HEK293T cells were first transfected with plasmids encoding HA-ATF6-mycHis or the point mutation variants. After 24 h, the cells were transfected with the control vector or a plasmid encoding Wt 3C tagged with FLAG. Cells were harvested and lysates were prepared 24 h post transfection. Equal amounts of cell lysates (30 μg/lane) were analyzed with 10% SDS-PAGE, followed by immunoblotting using antibodies against HA, FLAG, or GAPDH. The asterisk indicates the cleavage product N-70. The results shown here are representative of at least four independent experiments

**Fig. 6:**
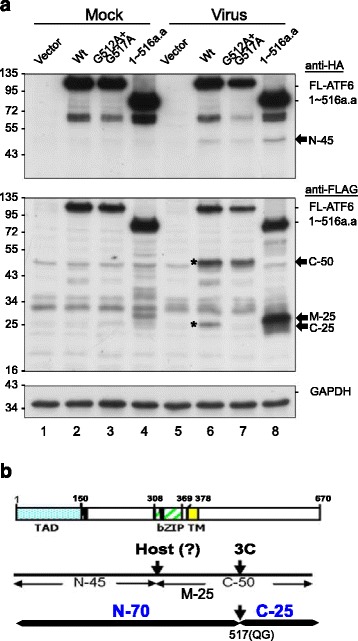
The cleavage of an interior proteolytic site of ATF6 was induced by virus rather than by 3C^pro^. **a** HEK293T cells were transfected with p90ATF6, a 512/517 double mutant, and a truncation mutant of amino acids 1–516 in AS3w-HA-FLAG for 24 h. After that, cells were infected with EV-A71 at an MOI of 200 and cell lysates were harvested at 16 h p.i. Equal amounts of protein lysates (30 μg) were analyzed by 10% SDS-PAGE followed by western blotting. The results shown here are representative of at least four independent experiments. **b** Schematic representation of the predicted cleavage of full-length ATF6 and amino acids 1–516 of ATF6. The arrows indicate the sites cleaved by 3C^pro^ and by an unknown protease induced by EV-A71 infection. The numbers indicate the predicted molecular weights of the products based on gel electrophoresis, and their relative positions are matched to the functional domains of ATF6

We proposed a scenario in which p90ATF6 is beneficial for viral replication, but it undergoes cleavage to generate p45ATF6. A prediction algorithm (NetPicoRNA 1.0) revealed several potential picornaviral protease cleavage sites in ATF6; thus, we examined the possibility of cleavage by the EV-A71-encoded proteases 2A^pro^ and 3C^pro^. Plasmids containing these proteins fused to a FLAG epitope were transfected into HEK293T cells. The 2A^pro^ and 3C^pro^ encoded by EV-A71 process the viral polyprotein to produce the mature and functional viral proteins, and they cleave cellular proteins such as eIF4G and CstF-64 [10, 24]. The viral proteases exhibited robust proteolytic activity, as shown by the presence of cleavage products of eIF4G and CstF-64 (indicated by arrows in the upper panels, Fig. 4a). Cells transfected with plasmids encoding 3C^pro^ but not 2A^pro^ exhibited ATF6 cleavage and the production of an amino-terminal ~ 70 kDa protein (designated N-70) and its potential corresponding C-terminal ~ 25 kDa protein (designated C-25) (lane 6, Fig. 4b-c). To examine the mechanistic basis of 3C activity, we generated a series of 3C^pro^ variants with mutations in one (H40D or C147S) or both (H40D/C147S) catalytic sites. The catalytic activity of all of these catalytic-dead mutant proteases was reduced to the background level, similar to that of the vector control, as demonstrated by their inability to cleave the CstF-64 substrate (lanes 1-5, Fig. 5a). A point mutant (R84Q) lacking RNA-binding activity but retaining protease activity was constructed as a control (lane 6, Fig. 5a). The point mutant and the singly and doubly catalytic-dead mutants showed similar expression levels to Wt 3C^pro^, as detected by anti-FLAG antibodies (Fig. 5b). These protease activity mutants, but not the RNA-binding mutant, lost the ability to cleave ATF6. This observation was confirmed by the appearance of N-70 fragments, demonstrating that the protease activity of 3C^pro^ was responsible for the cleavage of ATF6. We then mapped the 3C^pro^ cleavage sites in ATF6. Two potential 3C cleavage sites (glutamine-glycine; QG) were located at adjacent amino acids (511–512 and 516–517) near the C-terminus of ATF6 (Fig. 5c). The 3C cleavage sites were predicted to produce two major products with approximate masses of 70 kDa and 25 kDa, consistent with the western blotting data shown in Fig. 4. To confirm this prediction, both glycine residues in the potential cleavage sites were changed to alanine during a mutagenesis analysis. HEK293T cells were transfected sequentially with N-terminally HA-tagged ATF6 (HA-ATF6-FLAG) plasmids carrying these mutants and 3C^pro^; 24 h later, the cells were harvested for western blotting. The Wt ATF6 and the G512A mutant were susceptible to 3C^pro^ cleavage, which generated the N-70 fragment (indicated by asterisks in lanes 4 and 6 of Fig. 5d). Interestingly, the single G517A and double G512A/G517A mutants were resistant to cleavage, indicating that the second glutamine-glycine is the genuine cleavage site of EV-A71 3C^pro^. Together, these observations provide strong direct evidence that 3C^pro^ is the proteinase responsible for cleavage near the C-terminus of ATF6 during infection. Intriguingly, no cleavage products were appeared during viral infection in our previous findings [7]. We speculated that ATF6 may be cleaved by other mechanisms in addition to 3C or 2A cleavage. To explore this hypothesis further, we constructed a C-terminally tagged ATF6-truncated mutant comprising amino acids 1–516 [HA-ATF6(1-516 aa)-FLAG] that was devoid of the C-terminal 3C^pro^-cleaved product (Fig. 6). To examine the possible cleavage pattern during viral infection, we transfected HA-ATF6(1-516aa)-FLAG into HEK293T cells to improve the efficiency of ATF6 expression. A multiplicity of infection (MOI) of 200 was used because EV-A71 replicates less efficiently in HEK293T than in RD cells. A prominent N-terminal fragment ~ 45 kDa in size (N-45) was clearly detected by anti-HA antibodies in the virus-infected cells (lanes 6–8, upper panel, Fig. 6a). However, this fragment was not observed in the vector control (lane 5) or the mock-infected control (lanes 1–4). Two fragments, C-50 and C-25 (indicated by asterisks in lane 6, Fig. 6a), were produced from Wt ATF6, but only C-50 was detected in the G512/G517 mutant upon viral infection (lanes 6–7, middle panel). This finding was confirmed in a truncated mutant lacking the 3C^pro^ cleavage site, in which a smaller fragment (~ 25 kDa; designated M-25) was visible (lane 8). The M-25 fragment may have been present in the infected cells, but it was not detected due to a lack of specific antibodies. An additional cleavage site that generated an N-45 fragment was present in the middle of ATF6; this region is not cleaved by 3C^pro^ but is cleaved in a virus-inducible manner via an unknown mechanism, potentially by cellular proteolytic proteins. Based on the above findings, we created a diagram showing that EV-A71 infection generated three major fragments, namely, N-45, M-25, and C-25 (Fig. 6b). Collectively, the data demonstrated that the viral protease activity of 3C^pro^ was responsible for cleaving ATF6 to generate the N-70 and C-25 fragments. In addition, the N-45 fragment was generated in a 3C^pro^-independent but EV-A71-dependent manner; this fragment might have resulted from an as-yet-unidentified host proteolytic activity.

### Cellular caspase and XBP1-associated proteasome-dependent degradation did not play a role in the virus-induced decrease in ATF6

**Fig. 7:**
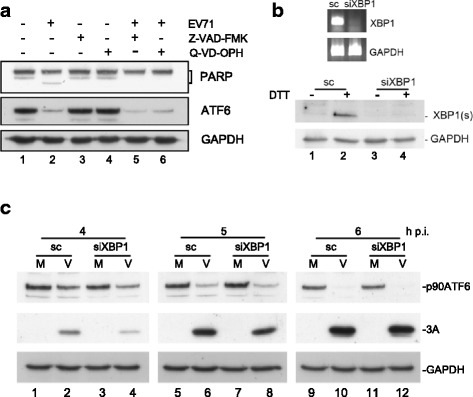
Effects of host proteinases and caspases on the virus-induced decrease in ATF6. **a** MCF7 cells were infected with EV71 and then treated with Z-VAD-FMK (20 μM) and Q-VD-OPH (20 μM) at 8 and 0 h p.i., respectively. Cell lysates were harvested at 10 h p.i. and evaluated by western blotting. **b** (Upper panel) Semiquantitative PCR analysis of XBP1 mRNA in RD cells transfected with siXBP1 or scrambled siRNA. (Bottom panel) RD cells were transfected with siXBP1 and then treated with 2.5 mM DTT, an ER stress inducer, for 6 h. The level of XBP1(s) was detected by western blotting. **c** RD cells were transfected with siXBP1 or scrambled siRNA for 72 h, infected with EV71 at an MOI of 10, and harvested at the indicated times after infection. The ATF6 levels in the virus-infected XBP1-silenced cells were detected by western blotting

Because N-45 was not produced by 2A^pro^ and 3C^pro^ cleavage, we next investigated the involvement of cellular factors in EV71-induced cleavage of ATF6. EV71 infection triggers apoptosis through the caspase pathway [11]. To determine whether the ATF6 fragment generated during EV71 infection in cells was caused by caspase activation, we infected MCF7 human breast cancer cells, which are deficient in caspase-3 protein levels and activity, in the presence or absence of caspase inhibitors (Fig. 7a). As expected, EV71 infection led to similar degrees of PARP (poly ADP-ribose polymerase) and ATF6 cleavage (lanes 1 and 2, Fig. 7a). However, the caspase inhibitors Z-VAD-FMK and Q-VD-OPH inhibited PARP cleavage but did not protect ATF6 from EV71 infection, demonstrating that the cleavage of ATF6 is not mediated by the caspase pathway (lanes 5 and 6, Fig. 7a). ATF6 undergoes proteasomal degradation, which is accelerated by the coexpression of unspliced XBP1 [29]. We therefore examined whether the knockdown of endogenous XBP1 would prevent XBP1-targeted ATF6 degradation. siXBP1 was transfected into RD cells for 72 h before viral infection (Fig. 7b-c). The endogenous levels of XBP1 mRNA and protein were reduced markedly (Fig. 7b), and the induction of XBP1 expression by DTT-induced ER stress was consequently inhibited in siXBP1 but not in scrambled transfected cells (lanes 2 and 4, bottom panel, Fig. 7b). A time course of infection revealed that the reduction in ATF6 became evident at 4 h p.i. and was more prominent with increasing length of viral infection in the scrambled and siXBP1-transfected cells (lanes 10 and 12, Fig. 7c). However, no protection against ATF6 cleavage was detected in the siXBP1-transfected cells. The expression of viral proteins, as represented by 3A expression, was slightly reduced in the siXBP1-transfected cells; however, this did not account for the cleavage difference (Fig. 7c).

## Discussion

We conclude that ATF6 plays a critical role in EV-A71 replication based on the following findings of this study: reduced ATF6 expression downregulated viral titers as a result of destabilized viral protein expression, and ATF6 was subject to proteolytic cleavage by viral 3C^pro^ and an additional virally induced cleavage that was 3C^pro^ independent. The cleavage effect was similar in three genotypes of EV-A71, including strain Tainan/4643/98 (isolated from a severe case; Fig. 1) [26], and in RD, HEK293T, MCF7, and glioblastoma SF268 cells (data not shown), suggesting that a reduction in ATF6 might not account for virulence or cell tropism. ATF6 is necessary and sufficient for viral protein stability. Is ATF6 also essential for host protein stability? Cell viability was not affected in siATF6-transfected cells, suggesting that ATF6 did not have a global effect on host proteins (data not shown). However, we cannot exclude the possibility that the stability of specific host proteins is affected by ATF6 knockdown. We also determined that the disappearance of ATF6 was not caused by cellular caspases or XBP1-associated proteasomes (Fig. 7) and proteinases (data not shown). Although we tested several proteinase inhibitors, including the serine proteinase inhibitors aprotinin and leupeptin (data not shown), we cannot exclude the possibility that the responsible proteinases were not identified in this study; some proteinase inhibitors can also inhibit viral replication when added at an early stage of viral infection [13]. Conversely, these agents can be added at a much later stage to prevent the inhibition of viral replication. However, at that point, the cleavage of ATF6 may have already occurred. Thus, it is paradoxical to use protease inhibitors to investigate the effects of cellular proteases on ATF6 cleavage.

We previously demonstrated that p90ATF6 disappeared, and a reporter assay showed that its downstream target chaperone genes were not activated during EV-A71 infection [7]. In the present study, we found that the disappearance of full-length ATF6 was caused by its proteolytic cleavage by the virus and, possibly, by the host. An N-terminal intermediate p45 fragment (N-45) derived from ATF6 was detected during EV-A71 infection, and this fragment migrated more rapidly than the p50 fragment produced by DTT treatment. These findings suggest that the conventional proteinases S1P and S2P, which are required for ATF6 processing during the UPR, are not directly involved in the generation of the N-45 fragment [15, 27]. This theory was supported by the observation that the serine proteinase inhibitor AEBSF, which acts against these site-specific proteases, did not alleviate the loss of the ATF6 signal (data not shown) [15]. We thus identified an aberrant ATF6 cleavage mechanism that differs from the conventional proteolytic processing of ATF6 mediated by site-specific proteases.

In cells transfected with full-length ATF6, the serial cleavage products N-45, C-50, and C-25 were detected during viral infection (Fig. 6). Antibodies specific to the middle fragment M-25 in infected cells are unavailable; thus, the presence of M-25 was inferred by detecting the FLAG-tagged truncated ATF6 construct (HA-ATF6(1-516 aa)-FLAG) (Fig. 6). The M-25 fragment was most likely produced by direct cleavage by 3C^pro^ at glutamine(517)–glycine(518) and by an EV-A71-induced protease with a predicted cleavage site between the TAD and the DNA-binding domain. Does cleavage first occur at G517, to be followed later by the other cleavages? The N-45 fragment was detected with both full-length ATF6 and the G517A mutant in virus-infected cells, indicating that the kinetics of cleavage is unlikely to be an ordered process. In addition, the detection of an apparent cleavage product, C-25, suggests that viral 3C^pro^ has an opportunity to cleave p90ATF6 (lane 6, Fig. 6). N-45 was detected during EV-A71 infection but not after 3C^pro^ or 2A transfection (Fig. 4), indicating that this cleavage site may be employed by cellular factors.

## Conclusions

Our collective results, particularly the finding that viral progeny production is inhibited in ATF6 knockdown cells, indicating that the cleavage of ATF6 by the host may offer an antiviral mechanism.

## Additional file

Additional file 1: Table S1. Primers used for plasmid construction. (DOC 40 kb)

## Abbreviations

ATF6: Activating transcription factor 6
CHX: Cycloheximide
CP: Cleavage product
CREB: Cyclic AMP-responsive element-binding protein
DMEM: Dulbecco’s modified Eagle’s medium
eIF2α: eukaryotic initiation factor 2α
ER: Endoplasmic reticulum
ERAD: ER-associated degradation
ERSE: ER stress response element
EV-A71: Enterovirus A71
FBS: Fetal bovine serum
FLuc: Firefly luciferase
GLS: Golgi localization sequences
IRE1: Inositol-requiring enzyme 1
MOI: Multiplicity of infection
p.i: Postinfection
PERK: PKR-like ER kinase
QG: Glutamine-glycine
RLuc: Renilla luciferase
S1P: Site-1 protease
S2P: Site-2 protease
sc: Scrambled control
siRNA: Small interfering RNA
TAD: Transcriptional activation domain
UPR: Unfolded protein response
Wt: Wild-type
XBP1: X-box-binding protein 1
