# Supplementary material for: A novel role of ER stress signal transducer ATF6 in regulating enterovirus A71 viral protein stability
Source: J Biomed Sci. 2018 Jan 31;25:9. doi: 10.1186/s12929-018-0412-x (PMC5793394; doi:10.1186/s12929-018-0412-x)
Supplement: Additional file 1: Table S1. — Primers used for plasmid construction. (DOC 40 kb) [file 12929_2018_412_MOESM1_ESM.doc]

Additional file 1: Table S1

| **Primers used for construction** | Forward primer | Reverse primer |
| --- | --- | --- |
| p90ATF6 in yT&A | 5' ATgggggAgCCggCTggggTTg 3' | 5’-TTgTAATgACTCAgggATggTgCTgACAACg 3' |
| HA-p90ATF6 in yT&A | 5’ agctaagcttaccatgtacccatacgatgttc  cagattacgctcttatgggggagccg 3’ | 5’ acgtgcggccgccttgtaatgactcagggatg |
| AS3W-HAp90ATF6-cFlag | 5' CAAgCTgCTAgCATgTACCCATACgATgTTCC 3' | 5' ggTACCgATATCAgTTgTAATgACTCAgggATgg 3' |
| AS3W-P1-cFlag | 5' gCATggCTAgCATgggCTCACAggTgTCCAC 3' | 5' CgAATgAATTCgTgAgAgTggTAATTgCTgTg 3' |
| AS3W-HAp90ATF6(G512A)-cFlag | 5' CAAgCTgCTAgCATgTACCCATACgATgTTCC 3'  5' gTA TTCTTCAggCTgCTCTggAAC 3' | 5' gTTCCAgAgCAgCCTgAAgAATAC 3'  5' ggTACCgATATCAgTTgTAATgACTCAgggATgg 3' |
| AS3W-HAp90ATF6(G517A)-cFlag | 5' CAAgCTgCTAgCATgTACCCATACgATgTTCC 3'  5' CTggAA CAggCCTCAAATTCTC 3' | 5' gAgAATTTgAggCCTgTTCCAg 3'  5' ggTACCgATATCAgTTgTAATgACTCAgggATgg 3' |
| AS3W-HA-ATF6-cFlag (1-516 a.a) | 5' CAAgCTgCTAgCATgTACCCATACgATgTTCC 3' | 5' ggTACCgATATCAgCTgTTCCAgAgCACCCTg 3' |
|  |  |  |
| AS3W-EV71 2A-cFlag | 5' gCATggCTAgCATggggAAATTTggACAgCAg 3' | 5' CgACTgAATTCgTCTgCTCCATggCTTCATC 3' |
| AS3W-EV71 3C-cFlag | 5' gATTAgCTAgCATggggCCgAgCTTggACTTC 3' | 5' CgACTgAATTCgTTTgTTCACTgCAAAAgTATC 3' |
| AS3W-EV71 3C(C147S)-cFlag | 5' gATTAgCTAgCATggggCCgAgCTTggACTTC 3'  5' gCAggACAgTCTggTggTgTTg 3' | 5' CAACACCACCAgACTgTCCTgC 3'  5' CgACTgAATTCgTTTgTTCACTgCAAAAgTATC 3' |
| AS3W-EV71 3C(H40D)-cFlag | 5' gATTAgCTAgCATggggCCgAgCTTggACTTC 3'  5' gCTCCCCAgAgACTCCCAAC 3' | 5' gTTgggAgTCTCTggggAgC 3'  5' CgACTgAATTCgTTTgTTCACTgCAAAAgTATC 3' |
| AS3W-EV71 3C(R84Q)-cFlag | 5' gATTAgCTAgCATggggCCgAgCTTggACTTC 3'  5' CgAAAAATTTCAggACATCAC 3' | 5' gTgATgTCCTgAAATTTTTCg 3'  5' CgACTgAATTCgTTTgTTCACTgCAAAAgTATC 3' |
| EV71 2231 IRES reporter (pcDNA-RHF 2231IRES) | 5’ggATCCTTAAAACAgCCTgTgggTTgC3’ | 5’ CTCgAgCCCATgTTTgATTg3’ |
